# IL-25 attenuates rheumatoid arthritis through suppression of Th17 immune responses in an IL-13-dependent manner

**DOI:** 10.1038/srep36002

**Published:** 2016-11-04

**Authors:** Dan Liu, Tuanping Cao, Na Wang, Chengfei Liu, Ning Ma, Ran Tu, Xiaoyun Min

**Affiliations:** 1 Core Research Laboratory, The Second Affiliated Hospital of Xi’an Jiaotong University, Xi’an, Shaanxi 710004, China; 2 Departement of Rheumatology and Immunology, Xi’an No. 5 Hospital, Xi’an, Shaanxi 710082, China; 3 Xi’an Institute of Rheumatology, Xi’an, Shaanxi 710082, China; 4 College of Life Sciences, Shaanxi Normal University, Xi’an, Shaanxi 710062, China

## Abstract

IL-25, a new member of the IL-17 cytokine family, is involved in type 2 immunity initiation and has been associated with the pathogenesis of rheumatoid arthritis (RA). However, its exact role remains unclear. Here, we aimed to analyse IL-25 expression in the serum and synovial fluid of RA patients and evaluated the correlations between serum IL-25 levels, clinical and laboratory values and inflammation cytokines. Additionally, we investigated whether IL-25 can suppress Th1/Th17 responses involved in RA pathogenesis. We further determined whether IL-25 can alleviate collagen-induced arthritis (CIA) development in mice and the underlying mechanisms using *in vitro* and *in vivo* experiments. Our results showed that IL-25 was upregulated in the serum and synovial fluid of RA patients. Increased serum IL-25 levels were associated with disease severity and inflammatory response in RA patients. Furthermore, IL-25 inhibited CD4^+^ T-cell activation and differentiation into Th17 cells, without affecting Th1 cells in human RA and CIA models. Administration of IL-25 could attenuate CIA development by Th17 suppression in an IL-13-dependent manner. Our findings indicate that IL-25 plays a potent immunosuppressive role in the pathogenesis of RA and CIA by downregulating Th17 cell response, and thus, may be a potential therapeutic agent for RA.

Rheumatoid arthritis (RA) is a prevalent common autoimmune disease characterised by chronic inflammation of the joint and synovial hyperplasia and progressive destruction of articular cartilage and bone, which seriously influences a patient’s quality of life^1^. Although the aetiology and pathogenesis of RA are still poorly understood, accumulating evidence has highlighted that an imbalance between pro- and anti-inflammatory cytokines maybe a key mechanism for disease progression in RA^2^. Therefore, identification of new cytokines offers promise in terms of the comprehensive understanding of the pathogenesis of RA and may potentially point to novel treatment targets.

Interleukin-25 (IL-25, also known as IL-17E), which was first identified by Fort *et al*. in 2001^3^, is a recently identified member of the IL-17 cytokine family^3^. The IL-17 family contains six members, from IL-17A to IL-17F, among which IL-25 has a unique structure and function, and the sequence of IL-25 is 16–20% similar to that of IL-17A^4^. IL-25 is produced by multiple cell types, including activated Th2 cells, bone marrow-derived mast cells, vascular endothelial cells, alveolar macrophages and basophils, as well as eosinophils^3^,^5^,^6^,^7^. Once secreted, IL-25 can bind to the receptors, IL-17RA and IL-17RB, and activate the NF-κB pathway where it plays the role of a Th2 cell-promoting cytokine^8^,^9^. IL-25 is believed to induce Th2 cell differentiation and response with the upregulation of Th2 cytokines, such as IL-4, IL-5 and IL-13^3^. The biological functions of IL-25 are markedly different from IL-17A and other IL-17 family cytokines. It promotes Th2 cytokine responses and plays a critical role in the development of allergic diseases^3^,^9^,^10^. On the other hand, IL-25 is reported to play an anti-inflammatory role in autoimmune and inflammatory diseases through the downregulation of Th1 and Th17 cell responses^11^,^12^,^13^.

Previous published studies have shown that IL-25 may be involved in the pathogenesis of RA. Gümüş *et al*.^14^ reported that serum IL-17A/IL-17E ratios were significantly higher in RA patients compared with osteoporosis patients and healthy controls. Wang *et al*.^15^ showed in an experimental collagen II-induced arthritis (CIA) model that IL-25 was increased in serum and inflamed knee joints in CIA mice. They also reported a negative correlation between IL-17 and IL-25 at the peak of CIA, suggesting that IL-25 might contribute to the pathogenesis of RA through decreasing Th17 responses. However, the exact role of IL-25 in the pathogenesis of RA remains to be elucidated.

In this study, we investigated the expressions of IL-25 in the serum and synovial fluid of RA patients and evaluated the correlation between serum IL-25 levels and clinical and laboratory values, as well as inflammation cytokines in RA patients. In addition, we investigated whether IL-25 is capable of suppressing Th1/Th17 responses, which are involved in the pathogenesis of RA. We further determined whether IL-25 can alleviate CIA development in mice and investigated the underlying mechanisms using *in vitro* and *in vivo* experiments. Our results demonstrated that IL-25 was upregulated in the serum and synovial fluid of RA patients. IL-25 ameliorated the CIA development by suppressing Th17 immune responses in an IL-13-dependent manner. To the best of our knowledge, this is the first study to provide evidence in both human RA and CIA mouse model that IL-25 is a critical anti-inflammatory cytokine in the pathogenesis of RA and may be considered as a potential therapeutic agent in the treatment of human RA.

## Materials and Methods

### Patients and specimens

A total of 48 RA and 40 osteoarthritis (OA) patients, from the Department of Rheumatology and Immunology, The No. 5 Hospital of Xi’an and The Second Affiliated Hospital of Xi’an Jiaotong University, were enrolled in this study. RA was diagnosed according to the 1987 revised criteria of the American Rheumatism Association^16^, and disease severity was assessed using the disease activity score 28 (DAS28)^17^. All RA patients were classified into three groups according to their DAS28: low (DAS28 < 3.2), moderate (DAS28 3.2–5.1) and severe (DAS28 > 5.1). Active disease was defined as DAS28 > 3.2. Fifty healthy volunteers (HCs) were simultaneously enrolled as controls. All the control subjects were matched with the patient population in terms of age and gender. Blood samples were obtained from a total of 48 RA patients, 40 OA patients and 36 HCs. Serum was obtained after centrifugation at 3000 rpm for 10 min; synovial fluid was collected from 48 RA and 40 OA patients and was centrifuged at 350 × g for 5 min. The serum and synovial fluid were collected and immediately stored at −80 °C until use. This study was approved by the Ethics Committee Board of The No. 5 Hospital of Xi’an and The Second Affiliated Hospital of Xi’an Jiaotong University. Informed consent was obtained from each participant according to the regulations of our Institutional Ethics Committee. All methods were carried out in accordance with the approved guidelines. The clinical characteristics of the patients and HCs are shown in Supplemental Table 1.

### Preparation of PBMCs and isolation of CD4^+^ T cells

Peripheral blood mononuclear cells (PBMCs) were isolated from the peripheral blood of RA patients and HCs with a Ficoll–Hypaque gradient (GE Healthcare Life Sciences, Little Chalfont, UK) following centrifugation. CD4^+^ T cells were isolated from fresh PBMCs using EasySep™ Human CD4^+^ T Cell Isolation Kit (STEMCELL Technologies, Vancouver, BC, Canada) according to the manufacturer’s protocol. The collected cells were washed and used for cell cultures.

### Cell culture and IL-25 treatment

Purified CD4^+^ T cells derived from PBMCs of RA patients or HCs were cultured at 1 × 10^6^/mL in RPMI 1640 medium containing 10% FBS in a humidified atmosphere of 5% CO_2_ at 37 °C. Cells were stimulated with or without plate-bound anti-CD3 (5 μg/mL) plus anti-CD28 (2 μg/mL) in the presence or absence of recombinant human (rh) IL-25 (100 ng/mL, R&D system, MN, USA) for 24 h. The supernatants were harvested and assayed for IL-17A, IFN-γ and IL-4 by enzyme-linked immunosorbent assay (ELISA); the remaining cells were collected for real-time PCR analysis. For CIA model, spleen CD4^+^ T cells were isolated from CIA mice using EasySep™ Mouse CD4^+^ T Cell Isolation Kit (STEMCELL Technologies, Vancouver, BC, Canada) according to the manufacturer’s instructions. Cells were stimulated with plate-bound anti-CD3 (5 μg/mL) plus anti-CD28 (2 μg/mL) in the presence or absence of recombinant mouse (rm) IL-25 (1, 10 and 100 ng/mL, R&D system, MN, USA) for 24 h. In some cases, anti-IL-4 mAb (10 μg/ml), anti-IL-5 mAb (10 μg/ml), anti-IL-13 mAb (10 μg/ml) or control IgG (10 μg/ml) were added to the cultures.

### CIA induction and IL-25 treatment

DBA/1J mice (age, 8–12 weeks) were purchased from Charles River Laboratories (Beijing, China) and maintained in a specific, pathogen-free animal facility at Shaanxi Normal University. All animal procedures were approved by the Institutional Animal Care and Use Committee of Xi’an Jiaotong University for the use of laboratory animals. CIA was induced according to standard protocol. Briefly, mice were immunised intradermally at two sites at the base of the tail with a total of 200 μL type II chick collagen (2 mg/ml; CII; Sigma-Aldrich) emulsified in complete Freund’s adjuvant (CFA) containing 5 mg/mL heat-killed *Mycobacterium tuberculosis* (H37Ra; Difco Laboratories, Detroit, MI, USA). On day 21, these mice were given a boost immunization of 80 μL CII dissolved in CFA. Beginning on day 1 after the second immunization, rmIL-25 (0.5 μg/mouse) was consecutively and intraperitoneally injected for 5 days. Mice were monitored daily for signs of arthritis. Clinical arthritis was evaluated using the following scale: grade 0 = no swelling; grade 1 = slight swelling and erythema; grade 2 = pronounced swelling; and grade 3 = joint rigidity. Mice were sacrificed on day 36; knee joints were dissected and processed for RNA extraction, as well as for histologic studies, and serum was collected for ELISA.

### ELISA

Human and mouse cytokine levels were measured using ELISA, according to the manufacturer’s instructions (eBioscience, San Diego, CA). Concentrations of CII specific IgG1 and IgG2a Abs (eBioscience) in the serum and synovial fluid of mice were measured using ELISA as previously described^18^,^19^. All samples were assayed in duplicate. The mean concentration was determined for each sample.

### RNA extraction and real-time PCR

Total RNA from the synovial tissue of CIA mice or culture cells was extracted with TRIzol Rreagent (Invitrogen) and reverse transcripted into cDNA. Transcription levels of IL-4, IFN-γ, IL-17A, ROR-γt, T-bet and GATA3 genes were analysed by real-time quantitative PCR using an ABI 7500 (Applied Biosystems) and SYBR green system (TaKaRa), according to the manufacturer’s protocol. The primer sequences are summarised in Supplemental Table 2. The relative gene expressions were normalised to the level of β-action and quantified by the 2^−ΔΔCT^ method. All reactions were performed in duplicate for each sample.

### Histopathologic analysis

Paraffin-embedded knee joint tissue sections of CIA mice (5-μm thick) were stained with hematoxylin and eosin. Histopathologic scoring of joint damage was performed under blinded conditions according to a scoring system widely used for evaluating synovitis, cartilage degradation and bone erosion^20^.

### Statistical analysis

Statistical analysis was performed using SPSS version 16.0 statistical software (SPSS, Chicago, IL, USA). The results are expressed as mean ± standard deviation (SD) or median with interquartile range (IQR). Clinical characteristics were compared using the chi-squared test for categorical variables; for continuous variables, the Kolmogorov–Smirnov test was employed to examine whether the acquired data were normally distributed. For nonparametric data, comparisons between the groups were performed using the Mann–Whitney U test or the Kruskal–Wallis test. One-way ANOVA or student t-test was used for parametric data. Spearman correlation test was used to evaluate the associations between serum IL-25 levels and laboratory values, as well as serum cytokine levels. P value < 0.05 was considered statistically significant.

## Results

### IL-25 is upregulated in serum and synovial fluid in RA patients

**Figure 1:**
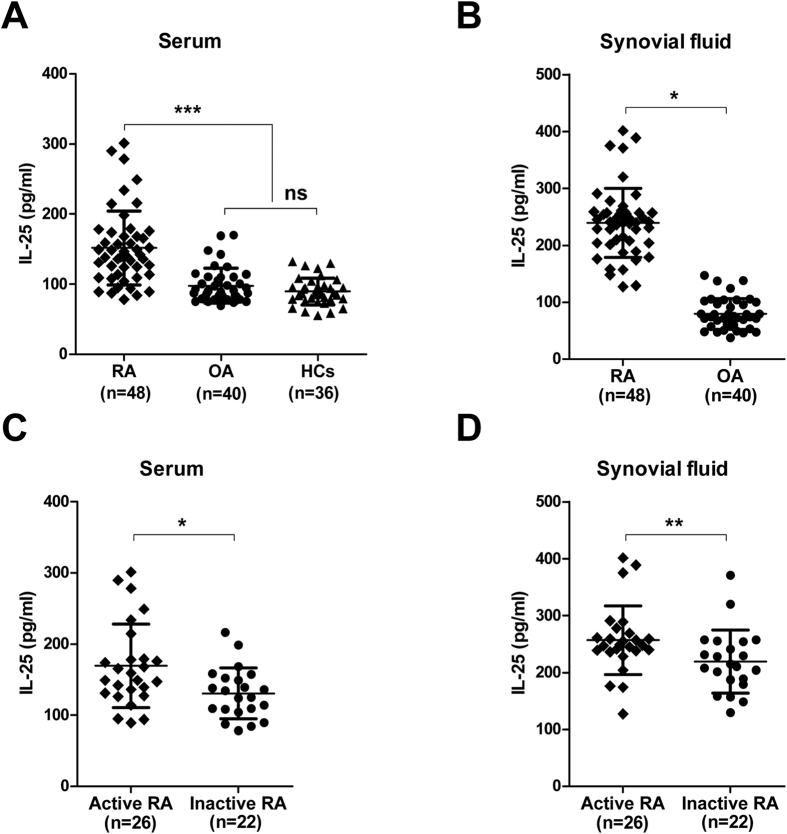
IL-25 is upregulated in serum and synovial fluid in RA patients. (**A**,**B**) Serum and synovial fluid IL-25 levels of patients with RA or OA or HCs by enzyme-linked immunosorbent assay (ELISA). (**C**,**D**) Serum and synovial fluid IL-25 levels in RA patients distributed according to disease activity (active, n = 26; inactive, n = 22) by ELISA. Data are presented as mean ± SEM. *P < 0.05, **P < 0.01, ***P < 0.001.

There is evidence that a broad range of cytokines is induced during the pathogenesis of RA, including the proinflammatory cytokines, IFN-γ, TNF-α and IL-17A, as well as type 2 cytokines, IL-4 and IL-13^2^. To determine whether IL-25 plays a regulatory role in RA, we first analysed the expression of this cytokine in RA patients. As shown in Fig. 1A,B, higher levels of IL-25 were detected in the serum and synovial fluid of RA patients than in those of OA patients and HCs. Additionally, patients with active RA (n = 26) showed higher serum (Fig. 1C) and synovial fluid (Fig. 1D) IL-25 levels than the inactive RA patients (n = 22). Taken together, these results suggest that IL-25 is upregulated in the serum and synovial fluid of RA patients, especially in active RA patients.

### Increased IL-25 levels are associated with disease severity and inflammation response in RA patients

**Figure 2:**
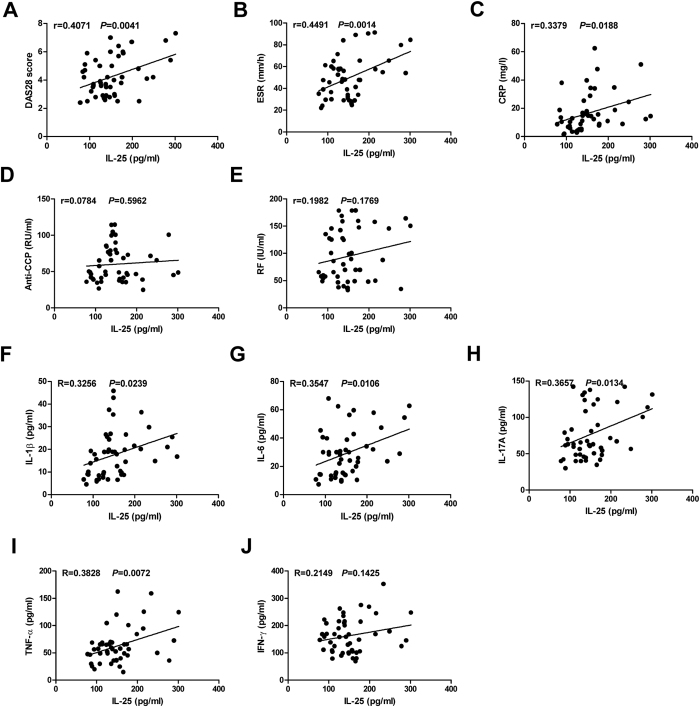
Increased IL-25 levels are associated with disease severity and inflammation response in RA patients. Correlation analysis between serum IL-25 levels and clinical and laboratory values as well as serum inflammation cytokines levels in RA patients. DAS28, 28-joint Disease Activity Score; ESR, erythrocyte sedimentation rate; CRP, C-reactive protein; RF, rheumatoid factor; anti-CCP, anti-cyclic citrullinated peptide antibodies. Spearman’s correlation test was used. The r value indicates the calculated regression coefficient.

Next, we examined the relationship between serum IL-25 levels and clinical and laboratory values, as well as inflammation cytokines, in RA patients. As shown in Fig. 2A–C, serum IL-25 levels were positively associated with DAS28 score, ESR and C-reactive protein. However, no significant correlation was observed between serum IL-25 levels and RF and anti-CCP antibody (Fig. 2D,E). Since pro-inflammatory cytokines, IL-1β, IL-6, IL-17A, TNF-α and IFN-γ, play an important role in promoting disease progression of RA, we assessed whether serum IL-25 levels in RA patients correlated with these cytokines. The results indicated that IL-25 serum levels positively correlated with the concentrations of IL-1β, IL-6, IL-17A and TNF-α, respectively (Fig. 2F–I). However, no significant correlation was observed between serum IL-25 and IFN-γ levels (Fig. 2J). Taken together, these results suggest that high levels of IL-25 may be associated with disease severity and inflammation response in RA patients.

### IL-25 inhibits Th17 cells without affecting Th1 cells in RA

**Figure 3:**
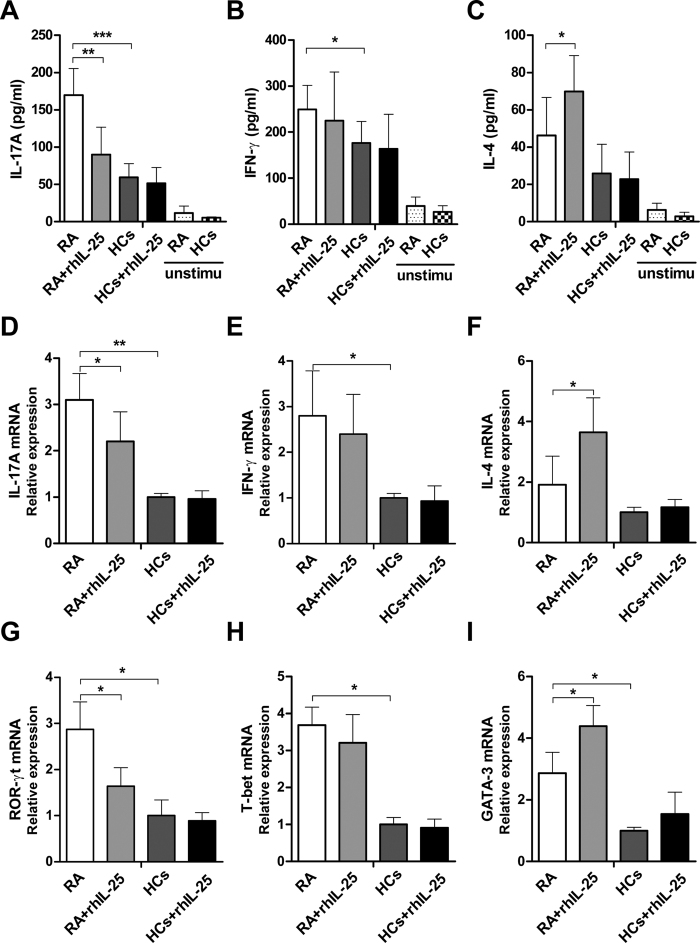
IL-25 inhibits Th17 cells without affecting Th1 cells in RA. CD4^+^ T cells from PBMCs of RA patients or HCs were isolated and stimulated with or without anti-CD3 (5 μg/mL) plus anti-CD28 (2 μg/mL) in the presence or absence of rhIL-25 (100 ng/mL) for 24 h. IL-17A (**A**), IFN-γ (**B**), and IL-4 (**C**) levels in supernatant were assessed by ELISA. The mRNA levels of IL-17A (**D**), IFN-γ (**E**), and IL-4 (**F**) were detected by real-time PCR. The mRNA levels of the master transcription factors ROR-γt (**G**), T-bet (**H**), and GATA3 (**I**) were detected by real-time PCR. Data are presented as the mean ± SEM of three independent experiments. *P < 0.05, **P < 0.01, ***P < 0.001.

IL-25 is a potent cytokine that drives the production of type 2 cytokines, such as IL-4, IL-5 and IL-13^3^, and it is reported to play an anti-inflammatory role in autoimmune and inflammatory diseases by downregulating Th1 and Th17 cell responses^13^. To investigate whether IL-25 has a similar capacity to suppress the Th1/Th17 responses involved in the pathogenesis of RA, CD4^+^ T cells from PBMCs of RA patients and HCs were isolated and stimulated with anti-CD3 (5 μg/mL) plus anti-CD28 (2 μg/mL) in the presence or absence of rhIL-25 (100 ng/mL). After 24 h of culture, supernatants were harvested and assayed for IL-17A, IFN-γ and IL-4; the remaining cells were collected for real-time PCR analysis. The results showed that CD4^+^ T cells of RA patients produced significantly higher levels of IL-17A and IFN-γ compared with HCs when stimulated with anti-CD3 and anti-CD28 (Fig. 3A,B,D,E). Treatment with rhIL-25 significantly reduced the expression of IL-17A in CD4^+^ T cells of RA patients, as determined using ELISA and real-time PCR (Fig. 3A,D), but IL-17A expression in HCs was not inhibited by treatment with rhIL-25. Interestingly, IL-25 treatment had no effect on IFN-γ expression (Fig. 3B,E), but promoted IL-4 production in RA patients (Fig. 3C,F).

To further elucidate whether IL-25 could regulate T helper cell subsets in RA, the mRNA levels were analysed for evaluation of the master transcription factors ROR-γt, T-bet and GATA3 that drive Th17, Th1 and Th2 differentiation, respectively. As shown in Fig. 3G–I, rhIL-25 treatment could markedly inhibit Th17 cell differentiation and promote Th2 cell differentiation in RA patients but failed to suppress Th1 cell differentiation. Taken together, these data suggest that IL-25 inhibits CD4^+^ T-cell activation and differentiation into Th17 cells, without affecting Th1 cells in RA.

### Administration of IL-25 attenuates CIA development in mice

**Figure 4:**
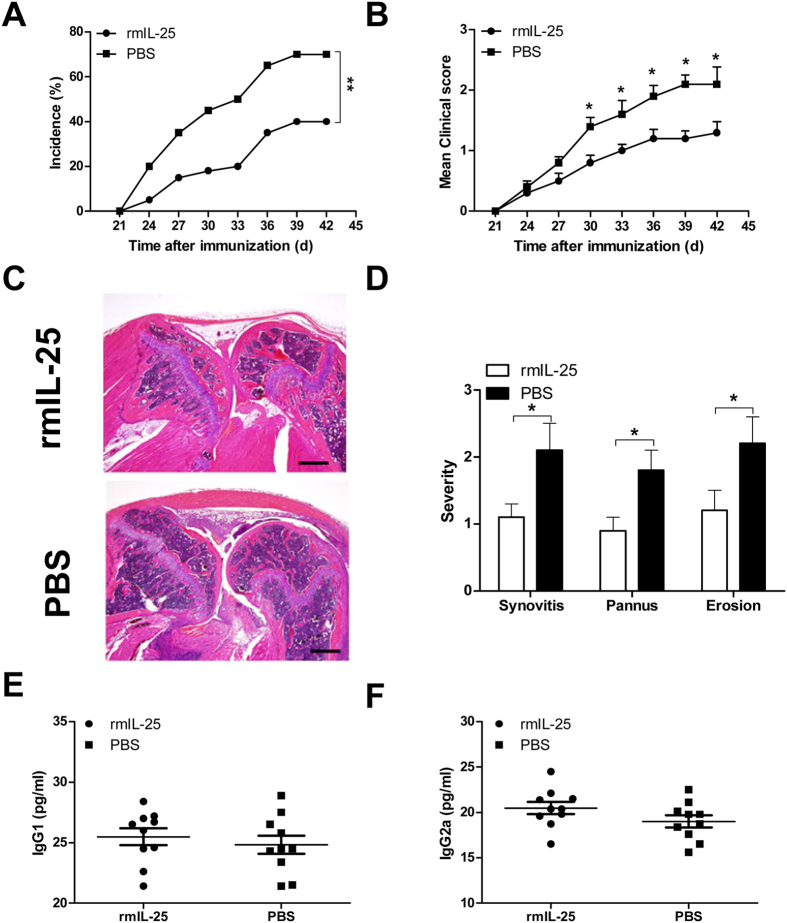
Administration of IL-25 attenuates CIA development in mice. Male DBA/1 mice were immunized with CII to induce arthritis, and received rmIL-25 (1 μg/mice) or PBS for 5 consecutive days beginning on day 1 after the second immunization with CII. Mice were killed on day 36 for experimental analysis. Incidence (**A**) and mean clinical scores (**B**) of CIA in mice treated with rmIL-25 or PBS (n = 10 per group). (**C**) Representative H&E staining of knee joints of rmIL-25 or PBS treated mice with CIA. Scale bars, 500 μm. (**D**) Evaluation results for synovitis, pannus, and erosion of bone and cartilage in the knee joint sections of rmIL-25 or PBS treated mice with CIA. (**E**,**F**) Serum levels of CII-specific immunoglobulin G2a (IgG1) and IgG2a antibodies were measured by ELISA. Data are presented as the mean ± SEM of three independent experiments. *P < 0.05, **P < 0.01, ***P < 0.001.

The above data suggest that IL-25 may play a potent immunosuppressive role in the pathogenesis of human RA. Thus, we next determined whether IL-25 can alleviate collagen-induced arthritis development in mice. Male DBA/1 mice were immunised with CII to induce arthritis and consecutively received rmIL-25 (1 μg/mice) or PBS for 5 days beginning on day 1 after the second immunization with CII. As shown in Fig. 4A,B, the RA incidence and symptoms of arthritis in IL-25-treated mice were significantly reduced compared with PBS controls. Histopathologic examination showed that the knee joints of IL-25-treated mice with CIA exhibited a significant reduction in synovial hyperplasia, cartilage damage and bone erosion compared with PBS controls (Fig. 4C,D). In addition, to evaluate whether the humoral immune response against CII was modulated by IL-25 treatment, serum from immunised IL-25 or PBS-treated mice were analysed for the presence of CII-specific IgG1 and IgG2a Abs. Interestingly, no obvious changes in the levels of serum CII-specific IgG1 and IgG2a Abs were observed in either group (Fig. 4E,F). Taken together, these results suggest that systemic administration of IL-25 attenuates arthritis onset and joint damage in CIA mice.

### IL-25 suppresses Th17 cell responses in CIA mice

**Figure 5:**
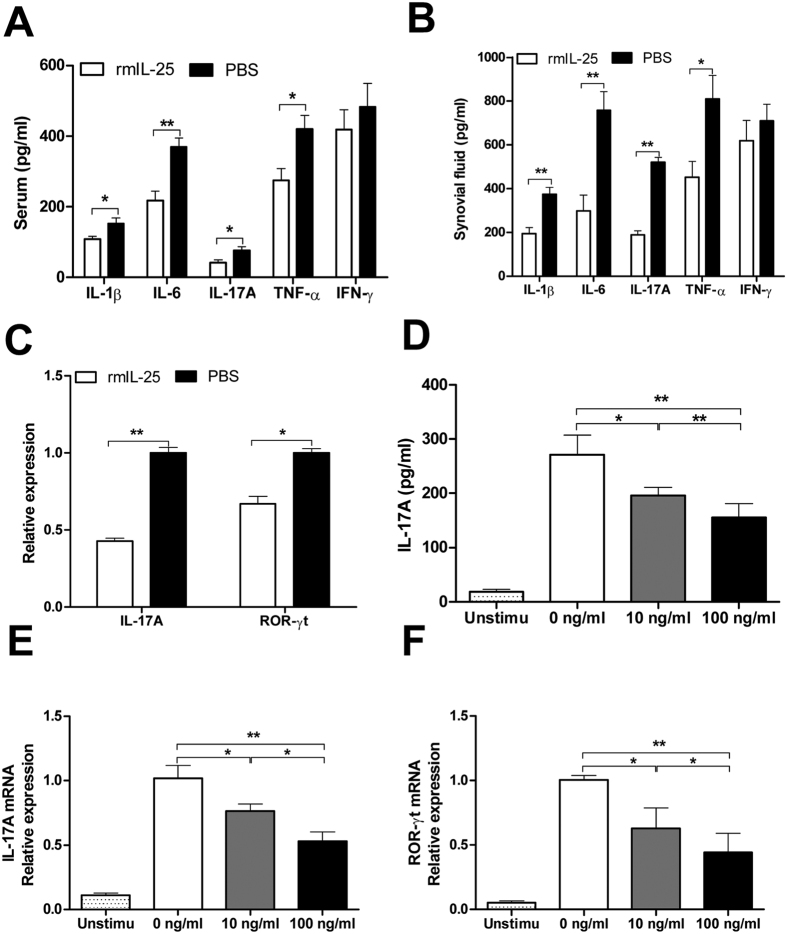
IL-25 suppresses Th17 cell responses in CIA mice. Serum (**A**) and synovial fluid (**B**) cytokines (IL-1β, IL-6, IL-17A, TNF-α, and IFN-γ) from CIA mice treated with rmIL-25 or PBS were measured by ELISA. (**C**) mRNA expression of IL-17A, and ROR-γt in synovial tissues of CIA mice treated with rmIL-25 or PBS were determined by realtime PCR. Spleen CD4^+^ T cells were isolated from CIA mice and stimulated with or without plate-bound anti-CD3 (5 μg/mL) plus anti-CD28 (2 μg/mL) in the presence or absence of rm IL-25 (1, 10, 100 ng/mL) for 24 h. IL-17A (**D**) levels in supernatant were assessed by ELISA. The mRNA levels of IL-17A (**E**), and ROR-γt (**F**) were detected by real-time PCR. Data are presented as the mean ± SEM of three independent experiments. *P < 0.05, **P < 0.01, ***P < 0.001.

To understand the mechanism by which IL-25 treatment reduced CIA, we first examined inflammatory cytokines in the serum of IL-25- or PBS-treated CIA mice. As shown in Fig. 5A, the serum levels of IL-1β, IL-6, IL-17A and TNF-α in IL-25-treated mice were markedly reduced compared with those in WT mice. In addition, the ELISA results showed that IL-25-treated mice had significantly lower amounts of IL-17 than PBS controls in synovial fluid (Fig. 5B); qRT-PCR also demonstrated that the transcription levels of IL-17A and ROR-γt were decreased in the synovial tissue of IL-25-treated mice compared with PBS-treated controls (Fig. 5C). To further characterise the Th17 cell response regulated by IL-25, spleen CD4^+^ T cells were isolated from CIA mice and cultured with or without rmIL-25 for 24 h to determine cytokine production. The results showed that rmIL-25 treatment significantly inhibited IL-17A production in a dose-dependent manner (Fig. 5D). In parallel, the mRNA expressions of key transcriptional factors for Th17 cells (ROR-γt), as well as IL-17A, also were decreased in a dose-dependent manner (Fig. 5E,F). In addition, we also observed dose dependent promoting effect of rmIL-25 on IL-4 production *in vitro* (data not shown), which was similar to the human RA results (Fig. 3C). Taken together, these results suggest that systemic administration of IL-25 could suppress Th17 cell response as well as IL-17-triggering cytokines in CIA mice.

### IL-25-mediated protection from CIA and Th17 suppression depends on IL-13

**Figure 6:**
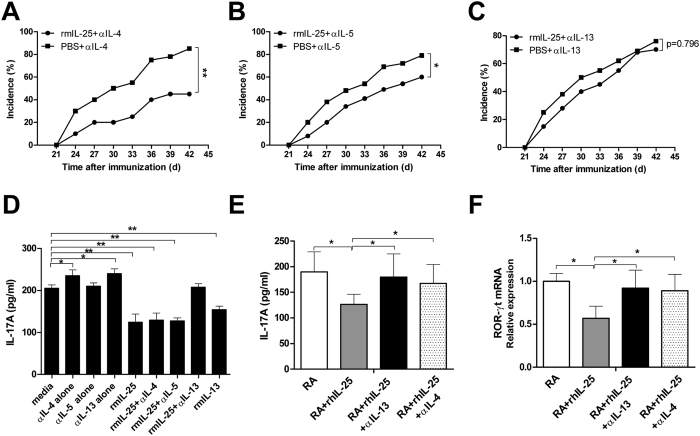
IL-25-mediated protection from CIA and Th17 suppression is IL-13 dependent. Male DBA/1 mice were immunized with CII to induce arthritis, and received rmIL-25 (1 μg/mice) or PBS for 5 consecutive days beginning on day 1 after the second immunization with CII, The mice were injected with anti-IL-4, IL-5 or IL-13 blocking monoclonal antibody (mAb) on day 3 before (−3d) and day 3 (+3d) after the second immunization with CII. Incidence of CIA in mice treated with anti-IL-4 (**A**), anti-IL-5 (**B**), and anti-IL-13 (**C**) (n = 8 per group). Spleen CD4^+^ T cells were isolated from CIA mice and stimulated with plate-bound anti-CD3 (5 μg/mL) plus anti-CD28 (2 μg/mL) in the presence or absence of rm IL-25 (100 ng/mL) for 24 h. anti-IL-4 mAb (10 μg/mL), anti-IL-5 mAb (10 μg/mL), anti-IL-13 mAb (10 μg/mL) or rmIL-13 (100 ng/ml) were added at the start of the culture. IL-17A (**D**) levels in supernatant were assessed by ELISA. CD4^+^ T cells from PBMCs of RA patients were isolated and stimulated with anti-CD3 (5 μg/mL) plus anti-CD28 (2 μg/mL) in the presence or absence of rhIL-25 (100 ng/mL) for 24 h. anti-IL-13 mAb (10 μg/mL) or anti-IL-4 mAb (10 μg/mL) was added at the start of the culture. IL-17A (**E**) levels in supernatant were assessed by ELISA. The mRNA levels of ROR-γt (**F**) was detected by real-time PCR. Data are presented as the mean ± SEM of three independent experiments. *P < 0.05, **P < 0.01, ***P < 0.001.

Following the observation that IL-25 attenuates CIA by suppression of Th17 responses, we performed *in vivo* and *in vitro* experiments to further investigate the underlying mechanism. Previous studies, as well as our own data shown here, indicate that IL-25 strongly induced type 2 cytokines, such as IL-4, IL-5 and IL-13 (Fig. 3C,F), which are potent CIA inhibitors. Therefore, we determined whether IL-25 suppression of CIA required the presence of these type 2 cytokines. Male DBA/1 mice were immunised with CII to induce arthritis and consecutively received rmIL-25 (1 μg/mice) or PBS for 5 days beginning from day 1 after the second immunization with CII. The mice were injected with anti-IL-4, IL-5 or IL-13 blocking monoclonal antibody (mAb) on day 3 before (−3d) and day 3 after (+3d) the second immunization with CII, and the incidence of arthritis was monitored. As shown in Fig. 6A,B, administration of IL-25 still exerted a protective effect from CIA on IL-4 mAb-treated mice and IL-5-mAb treated mice. However, IL-25 could not provide protection to IL-13 mAb treated mice (Fig. 6C). These data demonstrated that IL-13 plays an important role in IL-25-mediated suppression of CIA.

Following the observation that IL-25 exerted a protective effect from CIA that was mediated by IL-13, we used an *in vitro* cell culture system to further investigate the underlying mechanism. Spleen CD4^+^ T cells were isolated from CIA mice and cultured with rmIL-25 for 24 h. In this experiment, we found that the inhibitory effect of IL-25 on IL-17 production was completely abolished by the addition of anti-IL-13 mAb, whereas the addition of anti-IL-4 mAb and anti-IL-5 mAb had no effect (Fig. 6D). Moreover, addition of recombinant IL-13 to the cultures further decreased the production of IL-17, which was similar to the effects of adding rmIL-25 (Fig. 6D). We also confirmed this rescue effect in human CD4^+^ T cell culture conditions. As shown in Fig. 6E,F, neutralizing endogenous IL-13 or IL-4 by activated CD4^+^ T cells with anti-IL-13 mAb or anti-IL-4 mAb both resulted in an enhanced IL-17A production and ROR-γt mRNA expression in RA patients. Taken together, these results suggest that the CIA protection mediated by IL-25, as well as Th17 suppression, is IL-13-dependent.

## Discussion

The involvement of IL-25 in autoimmune diseases as an anti-inflammatory cytokine and an inhibitor of both innate and adaptive immunity has recently been reported^13^. In organ-specific autoimmunity, the balance of cytokines is a key determinant of resistance and susceptibility. In RA, disease susceptibility is thought to correlate with the expression of proinflammatory cytokines, such as IL-17, IFN-γ, TNF-α and IL-6. However, Th2 cytokines, such as IL-4 and IL-13, have been shown to play a relevant role in preventing or ameliorating inflammatory disease^21^. In the current study, we showed that IL-25, a Th2-promoting cytokine, was elevated in serum and synovial fluid of RA patients and associated with disease severity and inflammation response in RA patients. Moreover, IL-25 treatment had a protective effect against CIA development in mice, and this protection clearly depended on the suppression of Th17 immune responses. Therefore, the presence of IL-25 supports a cytokine environment that limits chronic inflammatory responses during the pathogenesis of RA.

Recent data demonstrated that IL-25 expression was increased in the serum of RA patients and CIA mice^14^,^15^. However, the expression and clinical significance of IL-25 in RA are not well known. Consistent with previous findings, our results also revealed higher levels of IL-25 in the serum and synovial fluid of RA patients compared with OA patients and HCs. Additionally, we clearly observed that IL-25 levels were significantly higher in patients with active disease than in patients with inactive disease. RA patients with severe and moderate disease activity had increased serum and synovial fluid levels of IL-25 compared with those patients with low disease activity or those in remission. Further analysis demonstrated that IL-25 expression in the serum of RA patients was positively associated with increased disease activity, including SJC, TJC, joint pain degree, HAQ score, ESR and C-reactive protein, as well as DAS28 score. Given that pro-inflammatory cytokines are involved in RA inflammatory response, a significantly positive correlation was observed between serum IL-25 levels and IL-1β, IL-6, IL-17A and TNF-α, respectively. These data suggest that pro-inflammatory cytokines are induced during the pathogenesis of RA and may in turn, stimulate anti-inflammatory cytokine IL-25 expression to downregulate excessive inflammation during the pathogenic process of RA. Interestingly, no significant correlation was observed between serum IL-25 and IFN-γ levels. Consistently, Wang *et al*. did not find any obvious correlation between IL-25 and IFN-γ in the spleen and knee joints of mice with CIA^15^, indicating that IL-25 had no direct effect on Th1-mediated factors. Taken together, our data suggested that IL-25 closely relates to RA, especially the disease activity of RA, and it may play a critical role in the pathogenesis of RA.

We then extended our studies to investigate the potential role of IL-25 in the induction of CD4^+^ T-cell activation and differentiation. IL-25 was found to markedly inhibit RA CD4^+^ T-cell activation to produce the proinflammatory cytokine IL-17A and promote IL-4 secretion. Consistently, IL-25 markedly inhibited the differentiation of RA CD4^+^ T cells into Th17 cells characteristic of the decreased expression of IL-17A and ROR-γt mRNA. Additionally, IL-25 promoted RA CD4^+^ T cells to differentiate into Th2 cells, which is characteristic of increased expression of GATA3 mRNA. However, this did not affect the expression of Th1-associated transcription factor T-bet. Consistent with our results in RA patients, we also observed similar results in CIA mice. These data suggest that in RA, IL-25 inhibits CD4^+^ T-cell activation and differentiation into Th17 cells, without affecting Th1 cells. In accordance with our findings, Benjamin *et al*. reported that IL-25 stimulation enhanced Th2 cytokine production but not that of IFN-γ in PBMCs of patients with Churg–Strauss syndrome^7^. In experimental autoimmune encephalomyelitis (EAE), neutralization of IL-17, but not of IFN-γ, in IL-25^−/−^ mice prevented EAE. In that study, it was reported that the ability of IL-25 to limit inflammation in CNS was independent of IFN-γ^11^. This finding indicated that IL-25 inhibited the development of Th17 cells by specifically promoting Th2 differentiation.

We subsequently attempted to confirm our findings of IL-25 as an anti-inflammatory cytokine in mice with CIA by systemic administration of rmIL-25 *in vivo*. As expected, administration of IL-25 in the CIA model resulted in a delayed onset of disease and alleviated the severity of clinical symptoms. Furthermore, the anti-arthritic effect of IL-25 in CIA was associated with a reduction in Th17 cell response, as well as IL-17-triggering cytokines. Notably, it has been reported that the effect of IL-25 may depend on its doses. Mchenga *et al*. found that, in dextran sulfate sodium-induced colitis, low dose (0.2 mg) aggravated, while high dose (0.8 mg) significantly attenuated colitis^22^. This phenomenon was also observed on Granulocyte macrophage colony stimulating factor (GM-CSF). GM-CSF is generally recognized as an inflammatory cytokine. Its inflammatory activity is primarily due its role as a growth and differentiation factor for granulocyte and macrophage populations^23^. GM-CSF-mediated inflammation has been implicated in some types of autoimmune diseases, including RA^24^. However, literatures also reveal that in many situations GM-CSF can act as an anti-inflammatory cytokine^25^. Low-dose GM-CSF induces tolerogenic DCs, and then selective promotes Treg cell expansion^26^. GM-CSF can suppress many autoimmune diseases such as experimental autoimmune thyroiditis^27^, Type-1 diabetes^28^, as well as myasthenia gravis^29^ though inducing natural Treg cells. Therefore, it should be consider the immunological status of the patient and progression of the autoimmune disorder in determining the potential clinical utility of IL-25.

Although the molecular mechanism of IL-25 in autoimmune disease remains unknown, previous studies, as well as the data reported herein, show that IL-25 strongly induced type 2 cytokines, such as IL-4 and IL-13^30^,^31^, which are potent CIA inhibitors. Therefore, we determined whether IL-25 suppression of CIA required the presence of these type 2 cytokines. Surprisingly, administration of IL-25 could still protect IL-4 mAb-treated mice and IL-5 mAb-treated mice from CIA, suggesting that IL-4 and IL-5 induced by IL-25 treatment are not sufficient for CIA suppression. In contrast to IL-4 and IL-5, we found that IL-13 is necessary for IL-25-mediated CIA suppression. Most interestingly, the blockade of IL-13 secretion by RA or CIA CD4^+^ T cells markedly attenuated the inhibitory role of IL-25 in modulating Th17 immune responses. These results are consistent with previous results that IL-13 is required for IL-25-mediated protection from EAE^11^. In addition, autoimmune diseases, including RA, are characterized by a deficit in central or peripheral tolerance, leading to an excessive immune responses and subsequent ongoing inflammation^32^. Treg cells play an important role in maintaining self-tolerance and immune homeostasis^33^,^34^. Recently study showed that IL-25 can promote the function of Treg cells^35^. Therefore, IL-25 may be beneficial for restoring and maintaining immune tolerance and subsequently stop the disease-causing immune attack on self-tissue in RA. Taken together, our findings suggest that IL-25 mediates CIA protection and Th17 suppression is dependent on IL-13. Nevertheless, further investigations are needed to investigate the detailed underlying mechanism.

In conclusion, the results of the present study provide evidence of the involvement of IL-25 in ameliorating the pathological process of CIA that is associated with the inhibition of Th17 immune response. Our findings indicate that IL-25 may be a potential therapeutic agent in the treatment of human RA.

## Additional Information

**How to cite this article**: Liu, D. *et al*. IL-25 attenuates rheumatoid arthritis through suppression of Th17 immune responses in an IL-13-dependent manner. *Sci. Rep*. **6**, 36002; doi: 10.1038/srep36002 (2016).

## Supplementary Material

Supplementary Information
