# Supplementary material for: IL-25 attenuates rheumatoid arthritis through suppression of Th17 immune responses in an IL-13-dependent manner
Source: Sci Rep. 2016 Nov 4;6:36002. doi: 10.1038/srep36002 (PMC5095710; doi:10.1038/srep36002)
Supplement: Supplementary Information [file srep36002-s1.doc]

**IL-25 attenuates rheumatoid arthritis through suppression of Th17 immune responses in an IL-13-dependent manner**

Dan Liu^1, 2, 3*^, Tuanping Cao^2^, Na Wang^1^, Chengfei Liu^1^, Ning Ma^1^, Ran Tu^4^, Ke Li^1^, Xiaoyun Min^1*^

^1^Core Research Laboratory, The Second Affiliated Hospital of Xi'an Jiaotong University, Xi'an, Shaanxi 710004, China.

^2^Departement of Rheumatology and Immunology, Xi'an No.5 Hospital, Xi'an, Shaanxi 710082, China.

^3^Xi'an Institute of Rheumatology, Xi'an, Shaanxi 710004, China.

^4^College of Life Sciences, Shaanxi Normal University, Xi'an, Shaanxi 710062, China.

* Corresponding author: Xiaoyun Min, or Dan Liu, Core Research Laboratory, The Second Affiliated Hospital of Xi'an Jiaotong University, No 157 Xiwu Street, Xincheng District, Xi'an, Shanxi 710004, China.

Tel. & Fax: +86-029-87678330

E-mail: dan_liu1988@sina.com

**Table S1. Characteristics of patients with RA, OA and healthy** controls.

| Characteristics | RA  (n=48) | OA  (n=40) | HCs (n=36) |
| --- | --- | --- | --- |
| Age (years) | 53.1 ± 13.2 | 54.5 ± 14.2 | 54.8 ± 15.4 |
| Gender (Male/Female) | 15/33 | 16/24 | 13/23 |
| Disease duration (years) | 4.3 ± 3.2 | - | - |
| DAS28 score | 4.8 ± 2.5 | - | - |
| ESR (mm/h) | 56.7 ± 35.9 | - | - |
| CRP (mg/l) | 31.9 ± 30.5 | - | - |
| RF (IU/ml) | 124.9 ± 104.8 | - | - |
| Anti-CCP antibody (RU/ml) | 72.9 ± 49.7 | - | - |
| Data are presented as Mean ± SD. TJC, tender joint count; SJC, swollen joint count; DAS28, disease activity score 28; ESR, erythrocyte sedimentation rates; CRP, C-reactive protein; RF, rheumatoid factor; Anti-CCP, anti-cyclic citrullinated peptide. | | | |

**Table S2. R**eal-time PCR primers

| Gene | Primer sequence (5’-3’) |
| --- | --- |
| hIL-4 | Forward: CGGCAACTTTGTCCACGGA |
|  | Reverse: TCTGTTACGGTCAACTCGGTG |
| hIFN-γ | Forward: TCGGTAACTGACTTGAATGTCCA |
|  | Reverse: TCGCTTCCCTGTTTTAGCTGC |
| hIL-17A | Forward: AGATTACTACAACCGATCCACCT |
|  | Reverse: GGGGACAGAGTTCATGTGGTA |
| hROR-γt | Forward: CTGGGCATGTCCCGAGATG |
|  | Reverse: GAGGGGTCTTGACCACTGG |
| hT-bet | Forward: GGTTGCGGAGACATGCTGA |
|  | Reverse: GTAGGCGTAGGCTCCAAGG |
| hGATA3 | Forward: GCCCCTCATTAAGCCCAAG |
|  | Reverse: TTGTGGTGGTCTGACAGTTCG |
| mIL-17A | Forward: TTTAACTCCCTTGGCGCAAAA |
|  | Reverse: CTTTCCCTCCGCATTGACAC |
| mROR-γt | Forward: GCGACTGGAGGACCTTCTAC |
|  | Reverse: CACATTCTGACTAGGACGACTTC |
| hβ-actin | Forward: CACCATTGGCAATGAGCGGTTCC |
|  | Reverse: GTAGTTTCGTGGATGCCACAGG |
| mβ-actin | Forward: GCACCACACCTTCTACAATGAG |
|  | Reverse: GCGTGAGGGAGAGCATAGC |
